# Estimated Glomerular Filtration Rate Decline Is a Better Risk Factor for Outcomes of Systemic Disease-Related Nephropathy than for Outcomes of Primary Renal Diseases

**DOI:** 10.1371/journal.pone.0092881

**Published:** 2014-04-02

**Authors:** Shuo-Chun Weng, Der-Cherng Tarng, Chyong-Mei Chen, Chi-Hung Cheng, Ming-Ju Wu, Cheng-Hsu Chen, Tung-Min Yu, Kuo-Hsiung Shu, on behalf of the CKDBHPDH investigators

**Affiliations:** 1 Center for Geriatrics and Gerontology, Taichung Veterans General Hospital, Taichung, Taiwan; 2 Division of Nephrology, Department of Internal Medicine, Taichung Veterans General Hospital, Taichung, Taiwan; 3 Institute of Clinical Medicine, National Yang-Ming University, Taipei, Taiwan; 4 Department and Institute of Physiology, National Yang-Ming University, Taipei, Taiwan; 5 Division of Nephrology, Department of Medicine and Immunology Research Center, Taipei Veterans General Hospital, Taipei, Taiwan; 6 Department of Statistics and Informatics Science, Providence University, Taichung, Taiwan; 7 Department of Biotechnology, HungKuang University, Taichung, Taiwan; 8 School of Medicine, Chung Shan Medical University, Taichung, Taiwan; 9 School of Medicine, College of Medicine, China Medical University, Taichung, Taiwan; UNIFESP Federal University of São Paulo, Brazil

## Abstract

**Background:**

Currently, the contribution of kidney function decline in renal and patient outcomes is unclear. There are few data on the associations of different etiologies of estimated glomerular filtration rate (eGFR) decline with outcomes in multidisciplinary care. The purpose of this investigation was to establish whether eGFR decline in patients with disease is an important risk factor for developing end-stage renal disease (ESRD) and death.

**Methods:**

From December 1, 2001 to December 31, 2011, 5097 adults with chronic kidney disease (CKD) received biochemical tests, physical examinations, a pathological examination, and a comprehensive questionnaire. We used linear regression models and multivariate Cox proportional hazards model to examine the outcome of eGFR decline in renal diseases with different etiologies.

**Results:**

Mean age was 68.1±16.1 (standard deviation, SD) years, and 63.3% patients were male. In the studied cohort, 58.2% of the patients had systemic disease-related nephropathy (SDRN), 29.4% had primary renal diseases (PRDs), and 12.4% had other etiologies. The eGFR decline in SDRN had a significant association with dialysis in the Cox proportional hazards model [crude hazard ratio (HR) = 1.07, 95% confidence interval (CI), 1.04 to 1.10; adjusted HR 1.05, 95% CI, 1.02 to 1.08]. Diabetic nephropathy (DN) had the most severe eGFR decline in CKD stages 3, 4, and 5, and all contributed to the initiation of dialysis and death regardless of whether DN with or without eGFR decline was considered to be the cause. Although hypertensive nephropathy (HN) was related to significant acceleration of eGFR decline, it did not lead to poor outcome. There were still discrepancies between eGFR decline and outcomes in PRDs, hypertensive nephropathy, and lupus nephritis.

**Conclusions:**

eGFR decline and CKD staging provide an informative guide for physicians to make proper clinical judgments in the treatment of CKD, especially SDRN. Poor control of the underlying systemic disease will thus lead to more rapid progression of SDRN.

## Introduction

The prediction of need for dialysis and risk of death in chronic kidney disease (CKD) patients has been shown to underestimate the importance of the rate of decline in renal function [1]–[4]. Although global guidelines have been proposed for estimating the need for preventive services for dialysis, in practice, they might not have been used for those purposes. Renal function is affected by both intrinsic mechanisms of renal disease (e.g., impaired auto-regulation [5]–[6], renal micro-inflammation [7]–[8], or limited renal functional reserve [9]) and extrinsic factors, such as hemodynamic changes, diabetes mellitus, hypertension, cardiovascular diseases or medication [6]. Physiological decline in kidney occurs due to the aging process, with approximately 10% of eGFR and 10% of renal plasma flow lost per decade after age 40 [10].

Systemic disease-related nephropathy (SDRN) more specifically refers to renal manifestations of systemic disease. A huge variety of systemic conditions can affect the function of the kidneys, from acute illnesses (including, for example, prolonged hypotension) to drugs and more insidious illnesses [2], [11], [12]. The highest prevalence of secondary glomerular diseases was diabetic nephropathy (44.3%) in the United States and systemic lupus erythematous (54.3%) in China [11]. Primary renal diseases include most common forms of glomerulonephritis, tubulointerstitial diseases, and microvascular or infectious etiologies without diabetic nephropathy, hypertensive nephropathy, lupus nephritis, congestive heart failure, human immunodeficiency virus (HIV) infection, liver disease, and dysproteinemias [11], [13], [17], [18]. It has been shown that extreme eGFR variation may occur in blacks with established CKD (from 1^st^ percentile, −23.6 mL/min/1.73 m^2^ per year to 99^th^ percentile, 18.5 mL/min/1.73 m^2^ per year), but annual change in eGFR was similar in all race groups with CKD (−3.7% to −4.3% per year) [3]. Biopsy-proved normo- and micro-albuminuric diabetic nephropathy (−4.9 ∼ −2.3 ml/min/1.73 m^2^ per year) [14], and overt diabetic nephropathy (−3.8±3.7 ml/min per 1.73 m^2^ per year) were reported in an observational retrospective study [15]. Renal function rates in lupus nephritis (LN) stratified by average urine protein excretion over time, i.e., 0–1, 1–2, and >2 g/day, respectively, were −1.15±5.37, 0.32±8.98, and −6.68±14.6 ml/min per 1.73 m^2^ per year [16]. For PRDs, the focal and segmental glomerulosclerosis (FSGS) displayed the highest incidence of ESRD (25.8%) and the fastest decline of eGFR (−4.6±17.6 ml/min per 1.73 m^2^ per year) [17]. The prognoses of SDRN and PRDs can be complicated by various modifiable risk factors, racial differences, glomerular hyperfiltration, interstitial fibrosis, tubular atrophy, and primitive etiologies [19]. However, relatively few studies have been conducted to establish whether the effects of different rates of eGFR decline, due to ordinary renal disease etiologies, on outcomes in the same cohort are dependent on SDRN and PRDs [14]–[20]. Therefore, in the present study, we investigated whether eGFR decline or diseases themselves have different pathological effects superimposed on physiological decline in the overall outcomes. Annual eGFR decline based on the coefficient of variation of the regression line is the most widely used method for estimation, and is used to show correlations with distinct histopathology and clinical diagnosis of CKD [1]–[6].

In this study, we investigated the effect of eGFR decline on the outcomes of different disease etiologies by conducting a prospective cohort study. The subjects were mostly middle-aged. We hypothesized that eGFR decline in patients with disease is an important risk factor for developing ESRD and death in cause-specific groups.

## Materials and Methods

### Study Population

**Figure 1:**
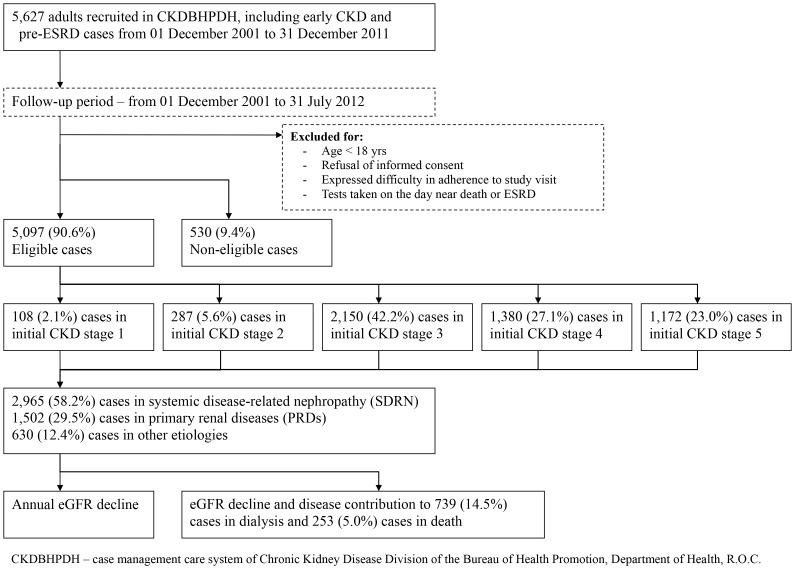
Study diagram of study cohort.

Using administrative data from the Chronic Kidney Disease division of the Bureau of Health Promotion, Department of Health, R.O.C. (CKDBHPDH), we identified records of men and women aged older than 18 years with CKD from five counties and cities in central Taiwan from 2001 to 2011. Study participants were recruited and followed up in three major hospitals under the Veterans Affairs Commission, Taiwan, namely, Taichung Veterans General Hospital (VGHTC; main institute), VGHTC Puli branch, and VGHTC Chiayi Branch. The referral centers included more than 4 million residents and the in-charge area was 10,660 square kilometers. The cohort database enrolled early CKD and pre-ESRD patients who were followed-up for longer than 6 months, from December 1, 2001, to July 31, 2012 (Figure 1). Reasons for disenrollment included initiation of dialysis, transfer to another hospital, all-cause mortality, and loss of contact. This study was approved by the institutional review board of Taichung Veterans General Hospital (No.CE12252). Although informed consent was required, the multidisciplinary care did not interfere with clinical decisions related to patient care. Whether written informed consent was given by participants (or next of kin/caregiver in the case of children) for their clinical records to be used in this study, every consent was obtained before the patient records/information was anonymized and de-identified prior to analysis.

### Study Design

Patients who had electronic medical records (visits for emergency department, outpatient clinic or hospitalization) in three hospitals were screened for potential recruitment. Screening for recruitment identified in previous units, the acute change in serum creatinine may have been affected by other factors during hospitalizations/emergency department presentations. We recruited the CKD cases under a relatively stable condition which may necessarily reflect their true eGFR change. To examine this question, we assembled a cohort with initial and subsequent eGFR using the 4-variable composite index (serum creatinine, age, race, and gender) – Modification of Diet in Renal Disease (MDRD) equation [1], [21]. The enrolled participants were allocated to five CKD groups, from stage 1 to stage 5. The eGFR decline (ml/min/1.73 m^2^ per year) with respect to time in different etiologies was analyzed by linear regression models. The coefficient of variation of the eGFR regression line accounts for the eGFR slope in repeated measurements [1]–[6]. This study considered three separate time intervals, 0–20, 20–40, and 40–60 months because the chosen cut-off points provided adequate statistical power for performing various subgroup analyses during the follow-up period. The cases with representative values of eGFR decline in each time interval excluded the cases that were censored when a value occurred outside the range of a measuring instrument. Such a situation can occur if an individual withdraws from the follow-up study, or if the individual is currently alive and on dialysis at the observational age.

A checklist providing information on socio-demographic characteristics, initial registration day, symptoms and signs of CKD, pre-existing comorbidity, current medication, and laboratory data requested from the Taiwan Society of Nephrology (TSN) for each patient, was completed by research nurses. Demographic information including age, sex, current smoking status, alcohol status, and malignancy were recorded. Diabetes mellitus, hypertension, and current medications were self-reported by the patients or retrieved from electronic records. Presence of cardiovascular diseases, i.e., coronary artery disease, stroke, congestive heart failure, arrhythmia, and peripheral arterial disease, was defined when identified in medical records. Body mass index (BMI) was calculated from the recorded height and weight. Serum creatinine was measured by the Jaffe method using a Beckman Synchron CX5 analyzer (USA) calibrated in accordance with the standards of the Chinese National Laboratory Accreditation program. Measurements of serum uric acid were obtained using the uricase method. Serum albumin was measured using bromocresol green (BCG) assay. Serum glycosylated hemoglobin A1c (HbA1c) was assessed by high-performance liquid chromatography (HPLC), and urine protein to creatinine ratio was measured by strip test. All biochemical laboratory tests were conducted by the Pathology and Laboratory Medicine Department of VGHTC. The time interval between the two eGFR tests or laboratory tests was required to be at least 3 months. Furthermore, we reviewed the medical records of clinical or pathological diagnoses of SDRN and PRDs partly with the assistance of our Clinical Informatics Research and Development Center. The primary outcome measures were incident ESRD warranting renal replacement therapy initiation and mortality due to eGFR decline based on etiological differences.

### Statistical Analyses

Our primary goal was to investigate the contribution of eGFR decline to the outcome of CKD patients with SDRN or PRDs after adjusting for age, sex, traditional risk factors (proteinuria, angiotensin-converting-enzyme inhibitor, ACEI/angiotensin receptor blocker, ARB, diabetes mellitus, and hypertension), and antilipemic agents in different stages of CKD [6], [19], [22]–[30]. For continuous variables, descriptive results were summarized by mean ± standard deviation and differences were tested by one-way ANOVA if the normality assumption was satisfied, or by Kruskall-Wallis test when the normal assumption was violated. For categorical variables, analyses were conducted using the Pearson χ^2^ test. Moreover, the *P*-value for trends was calculated using the Pearson correlation test when a variable was normal and using the Spearman’s rank test for continuous non-normal variables. Normality of continuous variables was tested with the Kolmogorov-Smirnov method. If the overall test for homogeneity was rejected, we further conducted pairwise comparison for post-hoc analysis to determine significant differences in risk factors among the five CKD groups.

For each patient, the eGFR decline rate (ml/min/1.73 m^2^) with respect to time in different etiologies was estimated by linear regression analysis for all outpatient measures of eGFR during different time intervals. If a patient had at least two outpatient eGFR measures in a specified interval, the eGFR decline for the corresponding etiology would be used.

We performed survival analysis to analyze the duration prior to events. Cox proportional hazards regression was used to determine the associations of eGFR decline in different CKD stages, except CKD stage 1 and 2, with initiation of chronic dialysis and all-cause mortality. Since early eGFR decline has less effect on the later outcome of CKD, this study investigated the contribution of eGFR decline in the last year of follow-up. Unadjusted rates were also reported because these represent actual etiological differences and the full burden of the diseases. Multivariate Cox proportional hazards model was conducted to assess the association of different eGFR decline rates as well as SDRN and PRDs with the primary endpoints, dialysis and death. A two-tailed *P* value <0.05 was considered statistically significant. Statistical analyses were implemented using **R** statistical software, version 2.15.3.

## Results

### Baseline Demographics

Participants’ characteristics were stratified by initial eGFR (Table 1). We also simulated the CKD cohort with the Chronic Kidney Disease Epidemiology Collaboration (CKD-EPI) formula which included variables such as age, sex, and serum creatinine, and concordance was found with a positive correlation between MDRD and CKD-EPI (Figure S1, Figure S2, Figure S3). Of the 5,097 patients included in the cohort, the mean age was 68.1±16.1 years. Patients with late-stage (stage 3, 4, 5) CKD were slightly older, more likely to have diabetes (CKD stage 3, 34.1%; stage 4, 42.3%; stage 5, 34.0%, *P* for trend <0.001), hypertension (CKD stage 3, 71.4%; stage 4, 70.5%; stage 5, 65.7%, *P* for trend <0.001), and hyperuricemia (CKD stage 3, 58.6%; stage 4, 67.8%; stage 5, 71.5%, *P* for trend <0.001). There was a high rate of prescription of patients on insulin (CKD stage 3, 9.3%; stage 4, 18.0%; stage 5, 13.6%, *P* for trend <0.001) and using an erythropoiesis-stimulating agent (ESA) in late-stage CKD patients (CKD stage 3, 2.2%; stage 4, 12.9%; stage 5, 47.9%, *P* for trend <0.001). Those with early-stage (stage 1 and 2) CKD were younger and smoking was less prevalent, but were more likely to have significant proteinuria. There were no differences in the distributions of cardiovascular disease, malignancy, abnormal body mass index (BMI) by gender, serum albumin, oral antidiabetics (OADs), and final death status among the five groups.

**Table 1:** Participants’ characteristics by different initial CKD stages.

	Stage 1	Stage 2	Stage 3	Stage 4	Stage 5	*P* value^a^
No. of participants	108	287	2,150	1,380	1,172	
Age (years)	38.6±16.7	52.2±17.7	67.5±14.9	67.2±14.8	63.5±14.6	<0.001^b^
Male gender (%)	46.3	57.1	74.8	59.9	48.9	<0.001^c^
Smoking history (%)	17.6	23.7	40.7	35.0	26.9	0.001^c^
Alcohol history (%)	53.7	42.9	25.2	40.1	51.1	<0.001^c^
Diabetes mellitus (%)	13.9	18.5	34.1	42.3	34.0	<0.001^c^
Hypertension (%)	25.9	47.4	71.4	70.5	65.7	<0.001^c^
Cardiovascular disease (%)	2.8	1.7	8.4	9.5	6.2	0.337^c^
Malignancy (%)	0.0	2.1	6.0	7.4	5.1	0.080^c^
BMI (kg/m2)†						
Male, abnormal (%)	71.2	61.7	65.4	61.8	61.4	0.094^c^
Female, abnormal (%)	55.4	59.8	71.6	70.3	59.5	0.085^c^
Average initial eGFR, MDRD (ml/min, median [IQR])	104.5 (95.3–113.0)	71.6 (65.3–79.2)	39.9 (35.1–44.4)	23.1 (19.1–26.6)	9.2 (6.5–12.0)	<0.001^d^
Average initial eGFR, CKD-EPI (ml/min, median [IQR]) ††	106.3 (100.2–117.0)	73.1 (66.3–81.7)	37.5 (32.9–42.6)	21.3 (17.6–21.2)	8.3 (5.8–10.8)	<0.001^d^
Urine PCR (mg/mmol, median [IQR])	2.9 (1.2–6.5)	1.5 (0.8–3.2)	0.5 (0.1–1.4)	1.0 (0.3–2.5)	1.5 (0.7–2.9)	<0.001^d^
Serum albumin <3.5g/dL, (%)	52.8	33.1	10.2	16.4	23.3	0.718^c^
Serum uric acid ≥7.2mg/dL, (%)	37.0	42.9	58.6	67.8	71.5	<0.001^c^
HbA1C						<0.001^c^
≥6.5 (%)	10.2	13.6	22.8	26.4	14.2	
<6.5 (%)	9.3	7.0	16.4	17.7	18.6	
NA (%)	80.5	79.4	60.8	55.9	67.2	
ACEI & ARB (%)	44.4	54.3	57	58.2	32.4	<0.001^c^
Insulin (%)	3.7	2.1	9.3	18.0	13.6	<0.001^c^
OAD (%)	13.0	16.0	31.3	38.0	24.7	0.105^c^
Antilipemic agents (%)	54.6	36.6	37.2	40.1	18.6	<0.001^c^
ESA (%)	0.9	0.7	2.2	12.9	47.9	<0.001^c^
Final status – Dialysis (%)	0.9	1.0	2.0	10.6	46.7	<0.001^c^
Final status – Death (%)	0.9	4.5	4.6	6.2	4.7	0.158^c^
Median follow-up in months (IQR)	20.3 (12.0–31.5)	27.5 (15.5–40.6)	27.7 (15.8–41.1)	25.7 (14.3–41.6)	16.0 (9.4–27.5)	<0.001^d^

Note: Data for categorical variables are given as percentage; data for continuous variables are given as mean ± standard deviation or median (interquartile range).

† The normal BMI value: male is 19.2–23.7 kg/m^2^ and female is 18.3–22.7 kg/m^2^ (Department of Health, Executive Yuan, Taiwan, R.O.C.). [Spindle 2009 health education advocacy plan survey summary report - The definition of body mass index in adults in Taiwan. February 2009 36(1) 23–26].

a For trend; ^b^By one-way ANOVA test; ^c^By Chi-square test; ^d^By Kruskal-Wallis test.

Abbreviations: BMI, body mass index; MDRD, Modification of Diet in Renal Disease equation; CKD-EPI, Chronic Kidney Disease Epidemiology Collaboration formula; PCR, urine protein to urine creatinine ratio; eGFR, estimated glomerular filtration rate; NA, non-available; ACEI & ARB, angiotensin-converting enzyme inhibitors & angiotensin II receptor blockers; OAD, oral antidiabetics; ESA, erythropoiesis-stimulating agent.

†† CKD-EPI formula, references:

1. Delanaye, P, Mariat, C. (2013) The applicability of eGFR equations to different populations. Nat. Rev. Nephrol 9: 513–522.

2. Levey AS, Stevens LA, Schmid CH, Zhang YL, Castro AF 3rd, et al. (2009) A new equation to estimate glomerular filtration rate. Ann Intern Med 150: 604–612.

3. Matsushita K, Mahmoodi BK, Woodward M, Emberson JR, Jafar TH, et al. (2012) Comparison of risk prediction using the CKD-EPI equation and the MDRD study equation for estimated glomerular filtration rate. JAMA. 307: 1941–1951.

After using pairwise comparison for post-hoc analysis, males were distributed equally in all CKD stages, except for 1,608 cases (74.8%) in CKD stage 3. With regard to lifestyle behaviors, never-smoking patients were more prevalent in early-stage CKD and the highest proportions of never-alcohol patients were observed in CKD stages 3 and 4. During follow-up, patients with CKD stages 4 or 5 were more likely to have dialysis (10.6% and 46.7%). But for the other rigorous outcome, all-cause mortality, there were no differences among the five groups. Furthermore, patients with CKD stage 5 had a shorter median follow-up in months (Table 1).

### Systemic Disease-related Nephropathy (SDRN) and Primary Renal Disease (PRD) Based on Initial Clinical or Pathological Diagnosis

Most SDRN was diagnosed by clinical judgment (96.1%). SDRN was diagnosed by pathological report in 3.9% of cases, but almost all cases of lupus nephritis were diagnosed by pathology. For entry eGFRs, diabetic nephropathy (DN) and hypertensive nephropathy (HN) were largely found in patients with relatively late-stage CKD (DN, stage 3, 29.0%; stage 4, 38.1%; stage 5, 32.1%, *P* for trend <0.001; HN, stage 3, 23.9%; stage 4, 19.2%; stage 5, 22.4%, *P* for trend 0.011, Table 2). Patients with diagnosis of PRDs included 32.8% cases diagnosed by renal-biopsy report and 67.2% cases that were identified in a search of index hospitals in Taiwan’s National Health Insurance Research Database (NHIRD). Several subgroups in PRDs had early-stage CKD, such as IgA nephropathy (IgAN) (stage 1, 14.8% and stage 2, 11.5% *vs.* other stages, *P* for trend <0.001), membranous nephropathy (MN) (stage 1, 16.7% and stage 2, 11.5% *vs.* other stages, *P* for trend <0.001), FSGS (stage 1, 3.7% and stage 2, 3.8% *vs.* other stages, *P* for trend = 0.012), and minimal change disease (MCD) (stage 1, 10.2% and stage 2, 3.5% *vs.* other stages, *P* for trend <0.001). Crescentic glomerulonephritis (GN) and rapidly progressive glomerulonephritis (RPGN) were found to be more prevalent in late-stage CKD (3, 4, 5). For other renal parenchymal diseases, most patients with chronic interstitial nephritis or rejection of kidney allograft had CKD stage 1, 2, or 3. Other etiologies, including obstructive nephropathy, urinary tract diseases, renal vascular diseases, hereditary diseases, and unknown causes were less prevalent in CKD stage 1 (Table 2).

**Table 2:** Diagnosed etiologies by different initial CKD stages.

	Stage 1	Stage 2	Stage 3	Stage 4	Stage 5	*P value* a
No. of participants	108	287	2,150	1,380	1,172	
Systemic disease-related nephropathy (n = 2,965, 58.2%)						
Diabetic nephropathy (%)	9.3	16.0	29.0	38.1	32.1	<0.001
Hypertensive nephropathy (%)	4.6	10.1	23.9	19.2	22.4	0.011
Lupus nephrophritis (%)	4.6	3.1	0.6	1.0	2.2	0.641
Others (%)††	0.0	2.8	5.6	5.4	3.4	0.034
Primary renal diseases (n = 1,502, 29.4%)						
IgA nephropathy (%)	14.8	11.5	3.1	3.4	1.6	<0.001
Membranous nephropathy (%)	16.7	11.5	1.5	0.8	0.6	<0.001
Focal segmental glomerulosclerosis (%)	3.7	3.8	1.8	1.8	1.3	0.012
Minimal change disease (%)	10.2	3.5	0.4	0.1	0.1	<0.001
Membranoproliferative glomerulonephritis (%)	0.0	0.7	0.2	0.4	0.1	0.524
Crescentic GN, RPGN (%)	0.0	0.0	0.1	0.2	0.6	0.002
Other renal parenchyma disease (%)*	33.3	26.5	21.6	18.0	20.7	0.001
Other etiology (n = 630, 12.4%)**						<0.001
	2.8	10.5	12.2	11.6	14.9	

a By Chi-square test.

†† Other systemic disease-related nephropathy included amyloidosis, scleroderma, multiple myeloma, gouty nephropathy, liver cirrhosis, heart failure, eclampsia, metabolic diseases causing renal failure, and other systemic disease causing renal failure.

*Other renal parenchyma disease of the primary renal diseases including chronic pyelonephritis, unrecovered acute renal failure, chronic glomerulonephritis, post-infectious glomerulonephritis, chronic interstitial nephritis, rejection of kidney allograft.

^**^Other etiology included obstructive nephropathy, urinary tract diseases, renal vascular diseases, hereditary diseases, other causes of renal failure, and renal failure with unknown causes.

### Different eGFR Decline Rates in Systemic or Primary Renal Etiology among Different Time Intervals

**Figure 2:**
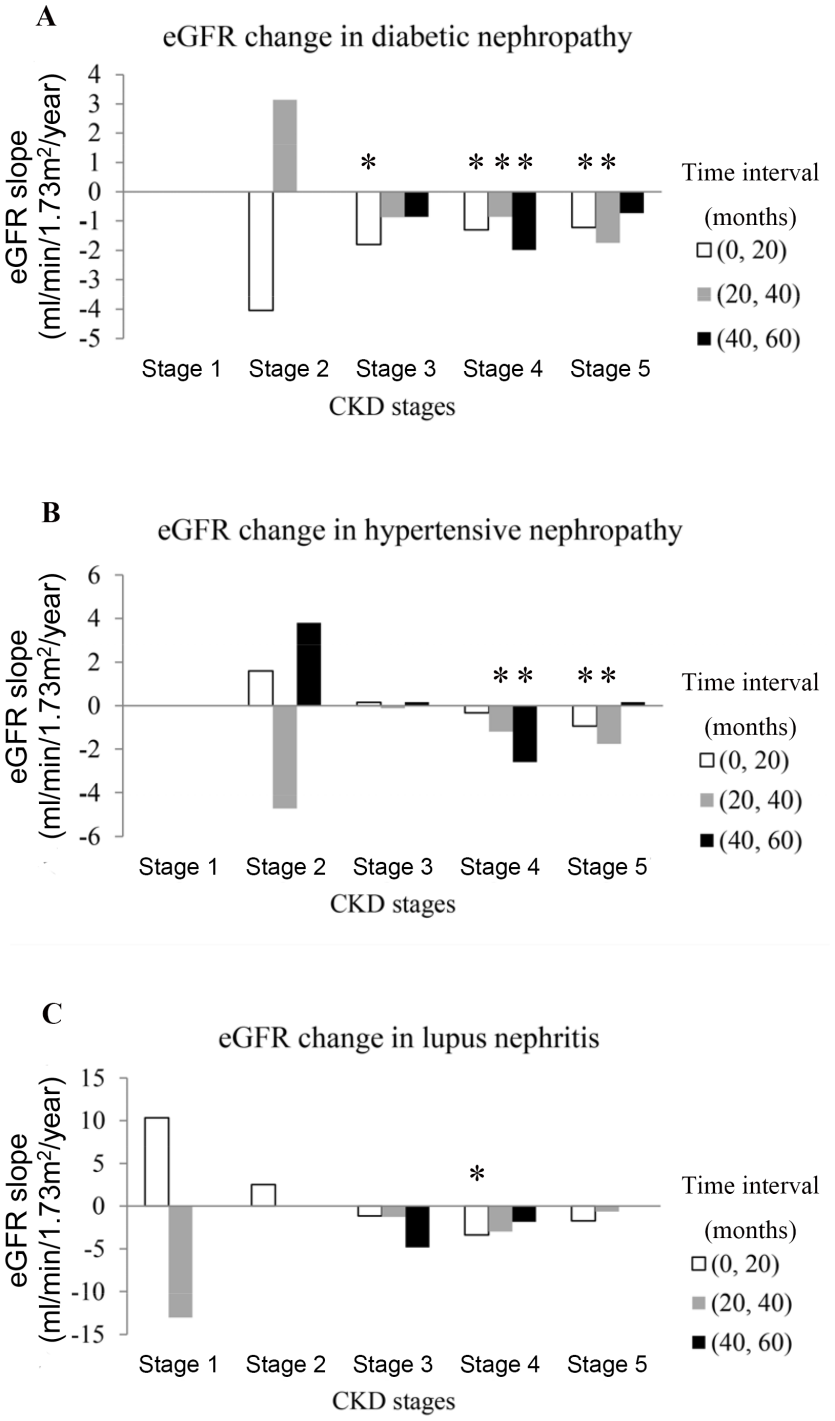
eGFR decline in systemic disease-related nephropathy (Significant codes: *, <0.05 means the eGFR decline is a significant value during this time interval under lineal regression model).

The data revealed the more skewed the linear regression line in disease etiology, the higher the variation in primitive eGFR. The importance of eGFR variation and decline can be seen in figure 2, which shows the serial eGFR changes in early-stage CKD (1 and 2) in SDRN during different time intervals was quite diverse. Rapid and significant eGFR decline was obvious in late-stage diabetic nephropathy (stage 3: −1.792 mL/min/1.73 m^2^ per year during 0–20th month, *P*<0.001; stage 4: −1.29 mL/min/1.73 m^2^ per year during 0–20th month, *P*<0.001, −0.867 mL/min/1.73 m^2^ per year during 20–40th month, *P* = 0.033, and −1.982 mL/min/1.73 m^2^ per year during 40–60th month, *P* = 0.023; stage 5: −1.208 mL/min/1.73 m^2^ per year during 0–20th month, *P* = 0.008 and −1.741 mL/min/1.73 m^2^ per year during 20–40th month, *P*<0.001, Figure 2A). There were also significantly faster rates of eGFR decline in the later stages of hypertensive nephropathy (stage 4: −1.197 mL/min/1.73 m^2^ per year during 20th –40th month, *P* = 0.006, −2.601 mL/min/1.73 m^2^ per year during 40th –60th month, *P* = 0.003; stage 5: −0.942 mL/min/1.73 m^2^ per year during 0–20th, *P*<0.001, −1.761 mL/min/1.73 m^2^ per year during 0–20th, *P*<0.001, Figure 2B). Lupus nephritis was not significantly associated with eGFR decline, except in CKD stage 4, with −3.384 mL/min/1.73 m^2^ per year during 0–20th, *P* = 0.027 (Figure 2C).

**Figure 3:**
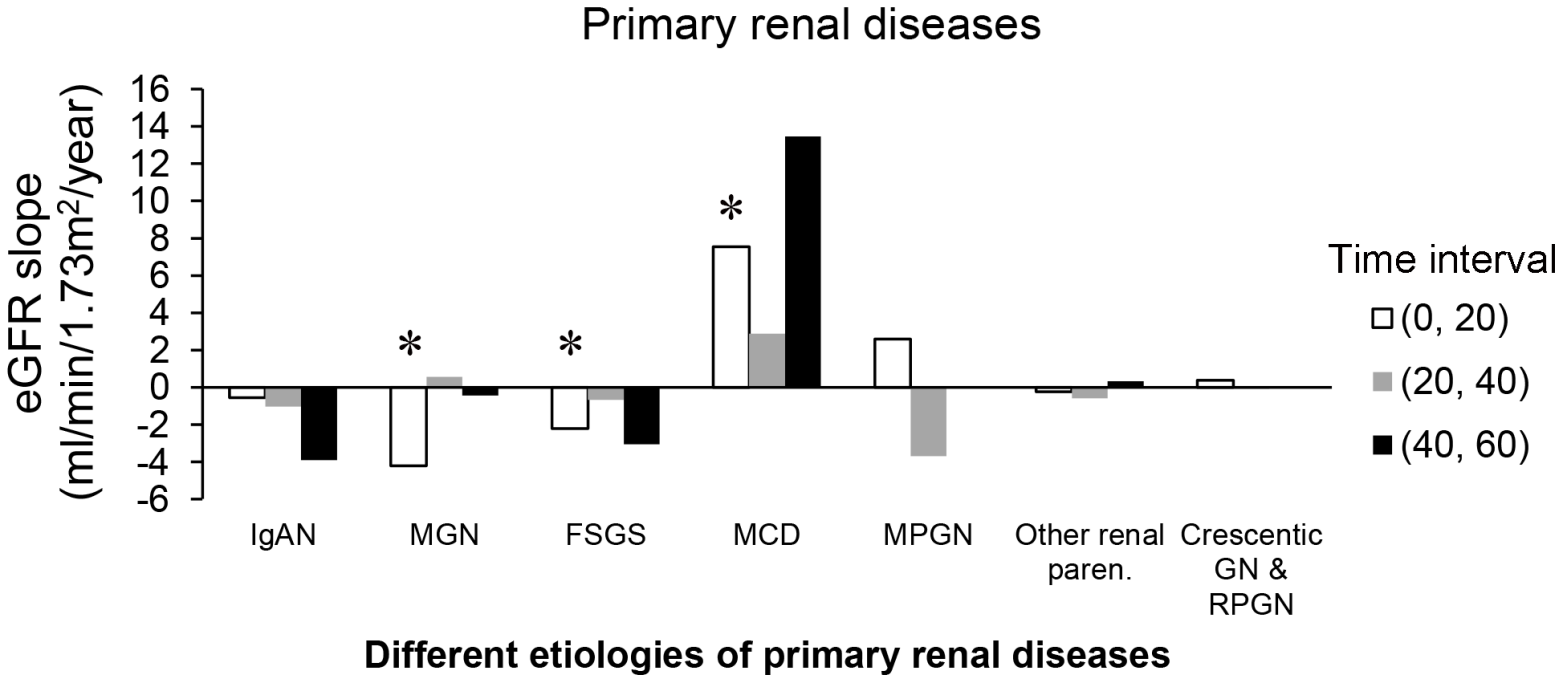
eGFR decline in primary renal diseases; IgAN, IgA nephropathy; MGN, membranous glomerulonephritis; FSGS, focal and segmental glomerulosclerosis; MCD, minimal change disease; MPGN, membranoproliferative glomerulonephritis; Other renal paren., other renal parenchymal disease; Crescentic GN& RPGN, crescentic glomerulonephritis and rapidly progressive glomerulonephritis (Significant codes: *, <0.05 means the eGFR decline is a significant value during this time interval under lineal regression model).

For PRDs, membranous nephropathy (MN) was associated with rapid eGFR decline in the early follow-up period (−4.210 mL/min/1.73 m^2^ per year during 0–20th month). Minimal change disease was correlated with significantly elevated eGFR in the early follow-up period (7.551 mL/min/1.73 m^2^ per year during 0–20th month). Focal segmental glomerulosclerosis (FSGS) was related to rapid eGFR decline in the early follow-up period (−2.203 mL/min/1.73 m^2^ per year during 0–20th month) (Figure 3).

### eGFR Decline Independently Predicted Poor Outcomes, Especially for SDRN in Maintenance of Dialysis

With regard to the primary endpoint, (434/2,723) 15.9% dialysis cases were stage 1–5 SDRN patients compared with (56/432) 13.0% dialysis cases who were stage 1–5 PRD (*P* = 0.113) (Table 3). The crude eGFR decline and adjusted (age, gender, eGFR decline, proteinuria, ACEI/ARB, and antilipemic agents) risk of dialysis and all-cause mortality in those with SDRN and PRDs are listed in Table 3. Several disease types categorized as SDRN (e.g., multiple myeloma) that could potentially be successfully treated were excluded. In addition, PRDs (e.g., Crescentic GN or RPGN, obstructive nephropathy, acute rejection in kidney transplant recipients, and pyelonephritis) with extreme values were excluded in the preliminary analysis.

**Table 3:** Association of eGFR decline with outcomes during study period by different disease etiologies.

	HR and 95% C.I. for dialysis	HR and 95% C.I. for death
	CKD stage 1–5	CKD stage 1–5
Systemic disease-related nephropathy, eGFR decline		
Number of events/Total numbers	434/2,723	164/2,723
Model 1	1.07 (1.04–1.10)***	0.95 (0.88–1.02)
Model 2	1.05 (1.02–1.08)***	0.99 (0.91–1.07)
Primary renal disease, eGFR decline		
Number of events/Total numbers	56/432	4/432
Model 1	1.01 (0.93–1.09)	1.02 (0.91–1.14)
Model 2	0.99 (0.92–1.08)	1.03 (0.75–1.43)

Model 1 is crude. Model 2 is adjusted for age, gender, eGFR decline, proteinuria, ACEI/ARB, and antilipemic agents.

Several disease types categorized as SDRN (e.g., multiple myeloma) that could potentially be successfully treated were excluded. In addition, PRDs (e.g., Crescentic GN or RPGN, obstructive nephropathy, acute rejection in kidney transplant recipients, and pyelonephritis) with extreme values were excluded in the preliminary analysis.

HR, hazard ratio; C.I., confidence interval.

Significant codes: *, <0.05; **, <0.01; ***, <0.001.

SDRN individuals with eGFR decline (per 1 ml/min/1.73 m^2^ per month) had a greater risk of initiating dialysis than that of PRDs (crude HR = 1.07, 95% CI = 1.04–1.10 and multivariate analysis, adjusted HR = 1.05, 95% CI = 1.02–1.08). That is to say, each unit of eGFR decline in SDRN increased the risk for dialysis by 5% after multivariate analysis. For the other outcome, (164/2,723) the mortality rate was 6.0% in stage 1–5 SDRN patients compared with (4/432) 0.9% in stage 1–5 PRDs (*P*<0.001), which was significantly different. However, there was no significant difference in the HR of eGFR decline to death between SDRN and PRD stages 1–5 in the analysis.

Table 4 presents an overall view of the average effects on outcomes (dialysis and all-cause mortality based on the most impactful CKD stages 3–5. eGFR declined independently and significantly increased the risk of poor outcome of dialysis in patients with CKD stages 3, 4, and 5 (Table 3B). However, only in CKD stage 4 did eGFR decline have a positive association with death after univariate and multivariate analysis. The effect of eGFR decline on death in patients with CKD stage 3 was observed to be inversely correlated with risk of death (HR = 0.87, 95% C.I. = 0.77–0.98, Table 4).

**Table 4:** Association of eGFR decline with dialysis and death in CKD stages 3∼5 cohort using univariate and multivariate cox proportional hazards analysis.

	HR and 95% C.I. for dialysis			HR and 95% C.I. for death		
CKD	Stage 3	Stage 4	Stage 5	Stage 3	Stage 4	Stage 5
Average eGFR decline (ml/min)	−0.115	−0.143	−0.272	−0.115	−0.143	−0.272
Number of events/Total numbers	42/2,150	146/1,380	547/1,172	98/2,150	86/1,380	55/1,172
Model 1	1.40 (1.24–1.58)***	1.79 (1.52–2.12)***	1.61 (1.44–1.80)***	0.87 (0.77–0.98)*	1.39 (1.01–1.89)*	1.61 (0.70–3.71)
Model 2	1.50 (1.30–1.73)***	1.60 (1.37–1.88)***	1.69 (1.49–1.92)***	0.91 (0.79–1.04)	1.69 (1.23–2.33)**	1.82 (0.62–5.32)

Model 1 is crude. Model 2 is further adjusted for age, gender, proteinuria, ACEI/ARB, antilipemic agents, diseases with a relatively large subgroup of patients (diabetic nephropathy, hypertensive nephropathy, IgA nephropathy, and membranous nephropathy).

HR, hazard ratio; C.I., confidence interval

Significant codes: *, <0.05; **, <0.01; ***, <0.001.

Table 5 shows the associations of disease etiologies and eGFR decline with dialysis and all-cause mortality in the cohort with CKD stages 3, 4, and 5 using multivariate Cox proportional hazards analysis. Regarding SDRN, diabetic nephropathy (DN) had the greatest effect on initiation of dialysis (model 1: CKD stage 3: HR = 3.04, 95% C.I. = 1.66–5.58; CKD stage 5: HR = 1.29, 95% C.I. = 1.08–1.55). DN also had the greatest contribution to initiation of dialysis after adjustment for traditional risk factors (model 2: CKD stage 3: HR = 5.06, 95% C.I. = 2.63–9.71; CKD stage 4: HR = 2.02, 95% C.I. = 1.42–2.87; CKD stage 5: HR = 1.51, 95% C.I. = 1.26–1.82). Furthermore, when additive eGFR decline was added to the model, there was a strong association between CKD stages of DN and dialysis. We also found a correlation of DN with additive eGFR decline to all-cause mortality among CKD stages 3,4, and 5 with the application of the same statistical analysis. A small increase in the percentage of dialysis in patients with hypertensive nephropathy (HN) was found after adjusting for conventional risk factors with additive eGFR decline. However, in CKD stage 5 in the HN group, there was a lower mortality rate (model 1: CKD stage 5: HR = 0.62, 95% C.I. = 0.33–1.26; model 2: CKD stage 5: HR = 0.47, 95% C.I. = 0.23–0.97; model 3: CKD stage 5: HR = 0.47, 95% C.I. = 0.23–0.97) after adjusting for eGFR decline and traditional risk factors. Multiple post hoc comparisons showed a greater prevalence of ACEI/ARB usage in patients with stage 5 CKD in the HN group compared with usage rates in the other groups.

**Table 5:** Associations of Disease Etiologies eGFR Decline with Dialysis and Death in CKD Stages 3∼5 Cohort Using Multivariate Cox Proportional Hazards Analysis.

	HR and 95% C.I. for dialysis			HR and 95% C.I. for death		
CKD	Stage 3	Stage 4	Stage 5	Stage 3	Stage 4	Stage 5
**Systemic disease-related nephropathy**						
DM nephropathy						
Model 1	3.04 (1.66–5.58)***	1.37 (0.99–1.90)	1.29 (1.08–1.55)**	1.58 (1.05–2.39)*	1.35 (0.88 – 2.07)	2.42 (1.42 – 4.12)**
Model 2	5.06 (2.63–9.71)***	2.02 (1.42–2.87)***	1.51 (1.26–1.82)***	1.77 (1.17 – 2.69)**	1.62 (1.05 – 2.50)*	2.93 (1.70 – 5.03)***
Model 3	5.20 (2.72– 9.96)***	1.91 (1.34–2.70)***	1.48 (1.23–1.78)***	1.74 (1.15–2.65)**	1.59 (1.03–2.46)*	2.87 (1.66–4.95)***
Hypertensive nephropathy						
Model 1	0.24 (0.07–0.77)*	0.99 (0.66–1.50)	0.91 (0.74–1.12)	1.23 (0.80 – 1.91)	1.04 (0.61 – 1.77)	0.62 (0.33 – 1.26)
Model 2	0.32 (0.10–1.07)	1.14 (0.75–1.74)	0.98 (0.80–1.20)	0.92 (0.59 – 1.43)	0.68 (0.39 – 1.16)	0.47 (0.23 – 0.97)*
Model 3	0.36 (0.11–1.18)	1.18 (0.77–1.80)	0.97 (0.79–1.20)	0.90 (0.57–1.41)	0.68 (0.40–1.18)	0.47 (0.23–0.97)*
Lupus nephritis						
Model 1	–	2.78 (0.88–8.73)	1.49 (0.86–2.59)	–	–	–
Model 2	–	1.47 (0.46–4.72)	1.32 (0.75–2.31)	–	–	–
Model 3	–	1.60(0.50–5.16)	1.31(0.75–2.31)	–	–	–
**Primary renal diseases**						
IgA nephropathy						
Model 1	2.91 (1.14–7.42)*	1.68 (0.85–3.29)	1.81 (1.07–3.08)*	–	–	–
Model 2	1.48 (0.55–3.93)	0.93 (0.46–1.89)	1.25 (0.73–2.14)	–	–	–
Model 3	1.43 (0.54–3.82)	0.89 (0.44–1.82)	0.90 (0.52–1.57)	–	–	–
MGN						
Model 1	2.39 (0.58–9.92)	0.74 (0.10–5.31)	1.43 (0.53–3.82)	1.1 (0.27 – 4.47)	–	–
Model 2	2.74 (0.65–11.56)	1.13 (0.16–8.12)	1.25 (0.47–3.36)	2.42 (0.58 – 10.09)	–	–
Model 3	2.28 (0.54–9.67)	1.22 (0.17–8.82)	1.34 (0.50–3.61)	2.54 (0.61–10.65)	–	–
FSGS						
Model 1	–	1.66 (0.68–4.06)	1.29 (0.64–2.59)	0.40 (0.06 – 2.88)	–	–
Model 2	–	0.95 (0.38–2.38)	0.99 (0.49–2.01)	–	–	–
Model 3	–	0.82 (0.33–2.08)	0.98 (0.48–1.98)	–	–	–
MCD						
Models 1 & 2 & 3	–	–	–	–	–	–
MPGN						
Model 1	–	2.63 (0.65 – 10.65)	–	–	–	–
Model 2	–	1.80 (0.44 – 7.36)	–	–	–	–
Model 3	–	1.81 (0.44 – 7.40)	–	–	–	–
Crescentic GN & RPGN						
Model 1	–	–	1.34 (0.50–3.57)	–	–	–
Model 2	–	–	1.17 (0.44–3.14)	–	–	0.96 (0.51 – 1.81)
Model 3	–	–	1.21 (0.45–3.25)	–	–	–
Other renal parenchymal diseases						
Model 1	0.80 (0.37–1.73)	0.86 (0.56–1.32)	0.82 (0.66–1.00)	0.89 (0.54 – 1.45)	0.78 (0.44 – 1.38)	0.94 (0.50 – 1.76)
Model 2	0.57 (0.26–1.25)	0.73 (0.48–1.12)	0.75 (0.61–0.92)*	0.96 (0.58 – 1.58)	0.85 (0.48 – 1.50)	–
Model 3	0.55 (0.25 – 1.22)	0.73 (0.48–1.13)	0.78 (0.63–0.96)*	0.99 (0.60–1.64)	0.88 (0.49–1.55)	0.95 (0.50–1.79)

Model 1 is crude for disease itself. Model 2 is further adjusted for age, gender, proteinuria, ACEI/ARB, antilipemic agents. Model 3 is further adjusted for all the items in model 2+ eGFR decline.

Significant codes: *, <0.05; **, <0.01; ***, <0.001.

For PRDs, IgA nephropathy had a greater effect on initiation of dialysis (model 1: CKD stage 3: HR = 2.94, 95% C.I. = 1.14–7.42; CKD stage 5: HR = 1.81, 95% C.I. = 1.07–3.08). However, no worse effects for CKD progression were observed after adjusting for conventional risk factors as well as the additive eGFR decline factors. Those non-available data were small sample size (Table 4); 95% confidence interval of hazard ratio is wide, which is the reasons for the data being inadequate for appropriate analysis. There was a large diversity of other renal parenchymal diseases and thus the data were difficult to interpret.

## Discussion

In this study, we found that eGFR decline independently predicted poor outcomes, especially the initiation of dialysis in systemic disease-related nephropathy (SDRN). For DN, each unit of eGFR decline had 5.20 times more risk for dialysis than that for other etiologies in CKD stage 3, 1.91 times more risk for dialysis than that for other etiologies in CKD stage 4, and 1.48 times more risk for dialysis than that for other etiologies in CKD stage 5 after multivariate analysis. The eGFR decline in primary renal diseases only makes a small contribution to the renal outcome and patients’ outcome in a unified and integrated health care system. This risk was independent of traditional risk factors (age, diabetes mellitus, proteinuria, baseline eGFR, and hypertension), and was recently shown to be a non-traditional risk factor reflecting both intrinsic and extrinsic factors in the kidney [2], [6], [19]. In this multi-center study we attempted to better understand the association of eGFR decline with the renal outcome and all-cause mortality. We found eGFR decline had a major effect on risk of dialysis and all-cause mortality after univariate and multi-variate analysis with specific primary renal disease etiologies (Table 3 and 4). This study demonstrated that eGFR decline rate, defined as ml/min per 1.73 m^2^ per year decrease, was associated with an increased risk for initiation of dialysis in SDRN, especially DN. Even after adjustment for age, the overall effect of eGFR decline beyond the normal aging process may be due to multiple co-morbidities, metabolic syndrome, and insulin resistance, which are risk factors for progression of CKD, and the rapid decline in renal function in the elderly [1].

One of the strengths of this study is that it is a long-term follow-up study spanning 10 years. In addition, a large sample size was analyzed which provided important information on the major etiologies of renal diseases. This study comprehensively examined long-term changes of eGFR in different renal diseases and showed associations of long-term outcomes with CKD. We explored the changes in eGFR over different time intervals in order to establish the relationships between longitudinal behavior of renal function and renal etiologies. Importantly, we investigated the contribution of eGFR decline (non-traditional risk factors) [2], [6], [19] in the final year of follow-up to the outcomes of CKD stages 3–5. Slopes were not calculated based on ordinary least squares as the renal outcome is technically problematic due to autocorrelation and heteroskedasticity. We did not adjust for baseline eGFR in slope models in order to avoid the so-called “horse-racing effect” [31].

Most predictors for ESRD and all-cause mortality found in ethnic Chinese cohorts showed similar findings in previous studies [32]–[36]. Taiwan is the only country to have achieved a hugely significant reduction in the incidence of ESRD in recent years, partly due to the inclusion of early CKD and pre-end-stage renal disease (ESRD) management programs in the National Health Insurance scheme [17], [32]. Despite the successful reduction of CKD, it must be remembered that there is a growing burden of CKD. In part this is due to the growth of other non-communicable diseases (NCDs), most notably diabetes. However, the emergence of new epidemics of CKD in the developing world, some of which appear to have environmental causes, has also contributed to the overall burden. Rapid decline of eGFR is a clinical concern for physicians and patients. GFR decline with aging is a sign of normal senescence, not disease [10]. However, among patients with similar levels of eGFR, clinical outcomes vary substantially by age. The well-known reasons for risk of rapid GFR decline are aging, racial difference (young blacks), albuminuria, hypertension, diabetes, and variable primary renal diseases [19]. However, the discrepancy between eGFR decline and clinical outcomes in long-term observational studies has yet to be fully elucidated.

To the best of our knowledge, the present study is the first to investigate the importance of eGFR decline and its differing contributions to rigorous clinical endpoints. However, several equivocal findings could be partly explained by different behaviors of medical practice. In clinical practice, eGFR decline in DN was kept under control in stage 2–4 CKD, but the improvements in stage 4 CKD of DN were lost after 40 months of follow-up. The decline of eGFR could be seen in stage 5 CKD in DN, and the decreased eGFR decline in the last period (40–60 months) was due to the ceiling effect of the lowest value of the eGFR (Figure 2A). Efforts to retard eGFR decline in stage 1–3 CKD in HN were effective, but no benefit was found after stage 4–5 CKD (Figure 2B). For PRDs, IgA nephropathy, FSGS, and memranoproliferative glomerulonephritis (MPGN) the decline rates were difficult to control (Figure 3). MN required more time to observe due to its chronic disease status and changes in eGFR were not obvious (Figure 3). In the episodic 12-month follow-up of eGFR decline, it was shown that multidisciplinary predialysis education (MPE) can be efficacious in motivated systemic-disease patients, especially for prolonging survival. Questions remain about its effectiveness (i.e., whether it works in everyday life) as it may be very hard to convince “non-compliant” patients. Skilled non-medical educators are often better than physicians at conveying information to patients, as they tend to have a more solid pedagogic and methodological background. There is no need for strong experimental, multidisciplinary care and non-randomized observational trials [36]. However, long-term follow-up studies are still worthwhile as they are capable of identifying potential risk factors.

In conclusion, eGFR decline was demonstrated to be superior to absolute GFR value as an indicator of kidney disease progression by the overall changes in kidney function over time. Diverse eGFR variations had better predictive values in systemic disease-related nephropathy than those in primary renal diseases. Diabetic nephropathy and hypertensive nephropathy were correlated with significantly greater eGFR decline in CKD stages 3, 4, and 5 compared with that of other disease etiologies. eGFR decline has a predictive value for dialysis and death in CKD stages 3, 4, and 5 in SDRN patients, especially DN. There were still discrepancies between eGFR decline and clinical outcomes in PRDs, hypertensive nephropathy, and lupus nephritis.

## Supporting Information

Figure S1 **Concordance was found with a positive correlation between MDRD and CKD-EPI.**
(TIF)

Figure S2 **The similar distribution of estimated GFR and eGFR-risk relationship were found between the CKD-EPI equation (red) and the MDRD Study equation (black) in our CKD cohort.**
(TIF)

Figure S3 **The positive correlation and distribution of eGFR between MDRD and CKD-EPI were shown by age categories (< and ≥65 years) in the CKD cohorts (female).**
(TIF)
