# Supplementary figures and images for: Estimated Glomerular Filtration Rate Decline Is a Better Risk Factor for Outcomes of Systemic Disease-Related Nephropathy than for Outcomes of Primary Renal Diseases
Source: PLoS One. 2014 Apr 2;9(4):e92881. doi: 10.1371/journal.pone.0092881 (PMC3973643; doi:10.1371/journal.pone.0092881)

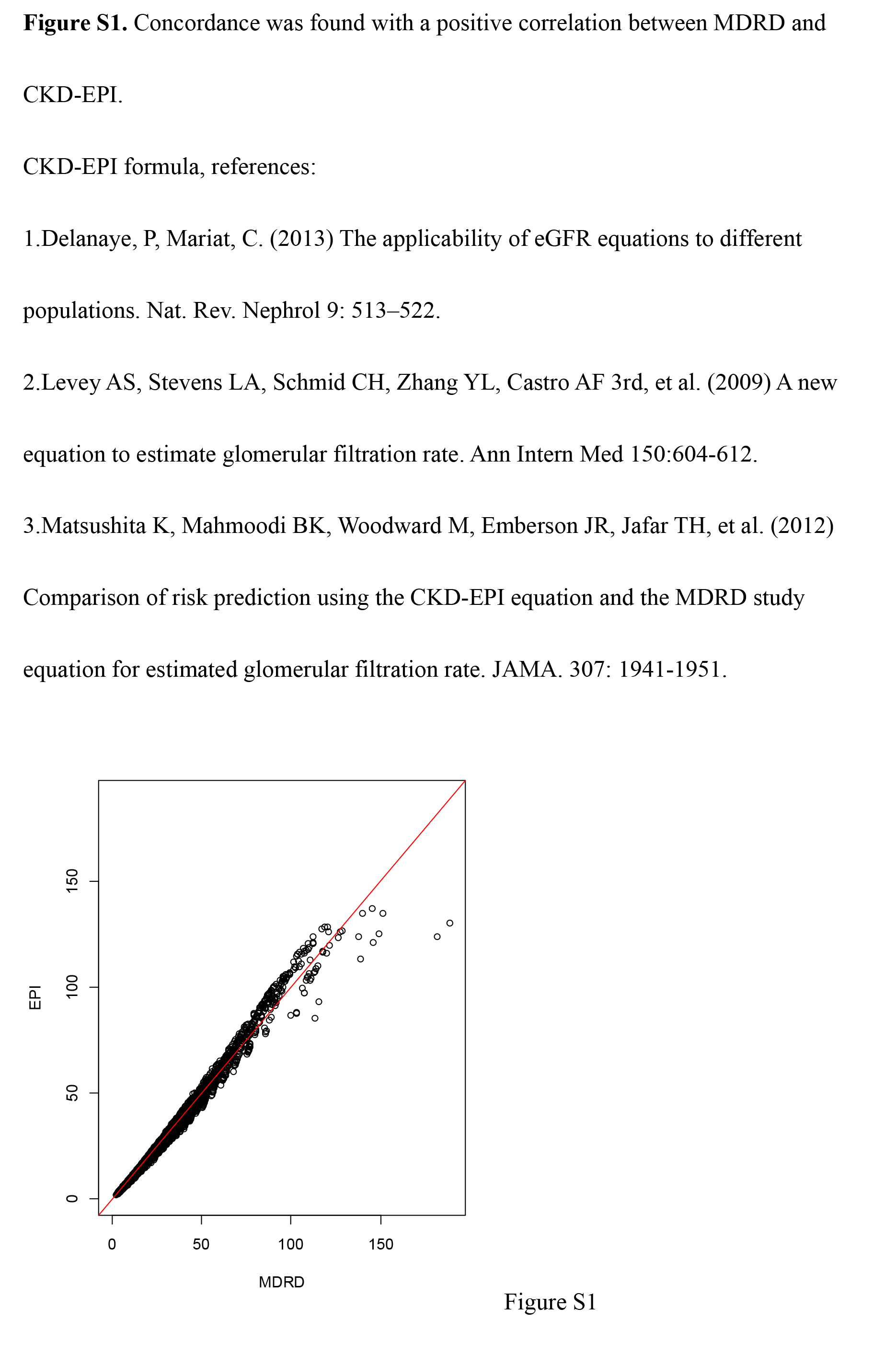

Supplement: Figure S1 — Concordance was found with a positive correlation between MDRD and CKD-EPI. (TIF) [file pone.0092881.s001.tif]

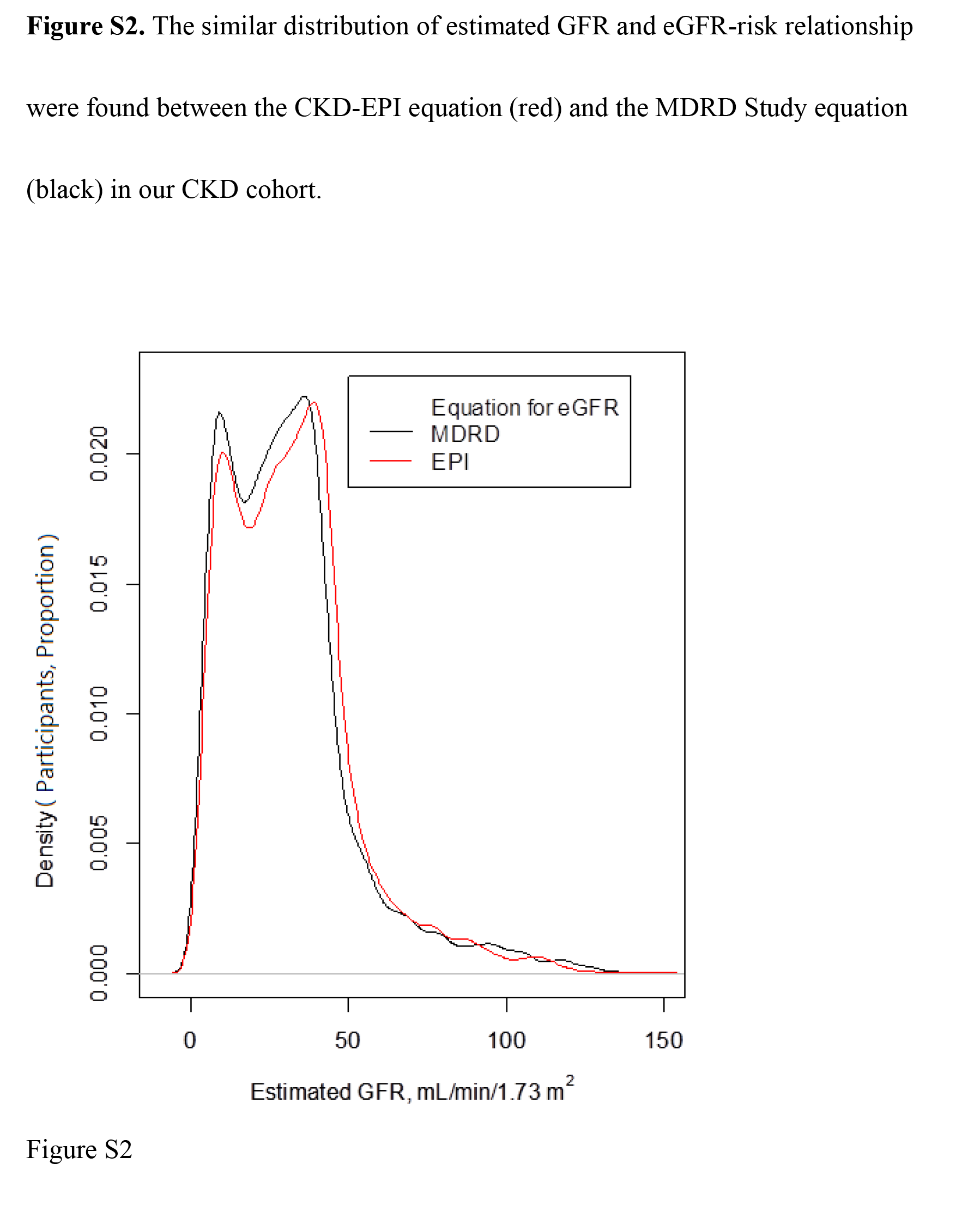

Supplement: Figure S2 — The similar distribution of estimated GFR and eGFR-risk relationship were found between the CKD-EPI equation (red) and the MDRD Study equation (black) in our CKD cohort. (TIF) [file pone.0092881.s002.tif]

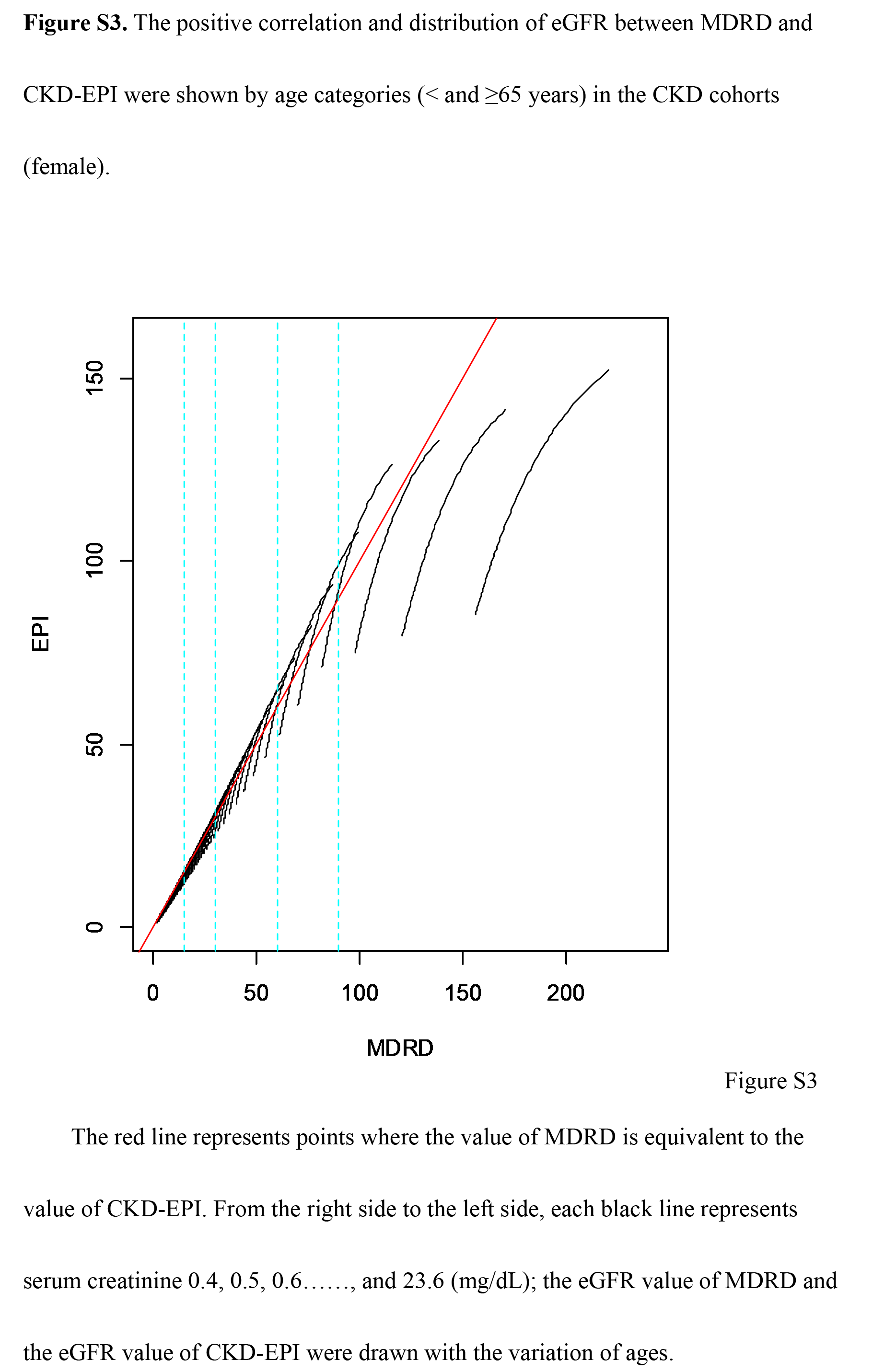

Supplement: Figure S3 — The positive correlation and distribution of eGFR between MDRD and CKD-EPI were shown by age categories (< and ≥65 years) in the CKD cohorts (female). (TIF) [file pone.0092881.s003.tif]
